# A systematic review and narrative synthesis of evidence from randomised controlled trials: the impact of behaviour guidance techniques on dental anxiety in paediatric patients

**DOI:** 10.1186/s12903-025-06533-x

**Published:** 2025-10-28

**Authors:** Josephine Kaur Dhaliwal, Mamdooh Alzyood, Reham Al Rawashdeh

**Affiliations:** 1 School of Human Sciences, Faculty of Education, Health & Human Sciences, University of Greenwich, Old Royal Naval College, Park Row, SE10 9LS London, United Kingdom; 2 School of Psychology, Oxford Brookes University, Social Work & Public Health, Tonge Building T.4.26, Headington Campus, OX3 0BP Oxford, United Kingdom; 3 Jordanian Ministry of Health Hospitals, Amman, Jordan

**Keywords:** Dental anxiety, Behaviour therapy, Oral health, Child, Behavioural medicine

## Abstract

**Aim:**

This review aims to evaluate the impact of various behaviour guidance techniques (BGTs) on reducing dental anxiety in paediatric patients, highlighting their effectiveness and areas for improvement.

**Method:**

A systematic review was conducted following PRISMA guidelines, focusing on randomised controlled trials published between November 2012 and July 2024 involving children aged 6–12 in dental settings. Relevant studies were identified through comprehensive searches in MEDLINE, EMBASE, Web of Science, PubMed, and the Cochrane Library, and analysed using narrative synthesis.

**Results:**

Eighteen studies met the inclusion criteria. Various BGTs effectively reduced dental anxiety in paediatric patients. Cognitive Behavioural Therapy (CBT), particularly as a distraction or self-help approach, was notably effective, alongside technology-based interventions like virtual reality (VR) and video modelling (VM). While sedation was also effective, CBT was preferred due to its lower risk of adverse effects.

**Conclusions:**

This review demonstrates that a range of BGTs, including CBT, VR, and aromatherapy, effectively reduce dental anxiety in children aged 6–12. CBT, particularly when combined with technology, was the most flexible and effective method. It provided both psychological and physical benefits with few risks. Sensory interventions, such as VR and aromatherapy, show promise in enhancing patient cooperation. Traditional methods like Tell-Show-Do (TSD) remain effective, but innovative, patient-centred techniques represent a shift in paediatric dental care. Future research should prioritise cost-efficiency and broader applicability in diverse settings.

**Clinical trial number:**

Not applicable.

## Background

Dental anxiety (DA) is common in children and is considered a normal part of development. It is also the most frequently encountered behavioural issue in paediatric dental care [1]. However, significant DA that causes avoidant behaviour can have a negative effect on a child’s oral health [2]. The prevalence of DA is estimated to be around 5.7–20.2% in children [3]. On average, 6 to 12 years was observed to be the most common age group in children to present with DA especially with dental caries [4]. Various scales can be used to assess prevalence of DA in children, such as Modified Child Dental Scale (MCDAS), Facial Image Scale (FIS) [5]. However, DA is usually self-reported, or parent reported [6].

Behaviour guidance acts as a continuum of interaction among the oral health professionals, the patient and the family while ensuring the safety of both the dental team and the child during medically essential care was introduced by the American Academy of Pediatric Dentistry (AAPD) [7]. Traditional techniques include protective stabilisation, such as using a papoose board, or pharmacological methods like sedation or general anaesthesia. These are not routine treatments and are typically taught only in advanced paediatric dentistry programmes [8]. More traditional methods include direct observation, nonverbal communication, distraction, memory restructuring and enhanced control. Newer techniques like Sensory-adapted dental environment (SADE), Picture exchange communication (PECS), Animal-assisted therapy (AAT), Virtual Reality (VR) are based on CBT model developed by Williams and Garland [9]. In alignment with the current recommendations from the American Academy of Pediatric Dentistry (AAPD), this review adopts the term ‘behaviour guidance techniques’ (BGTs) in place of the older term ‘behaviour management techniques.’ This terminology reflects a more supportive and collaborative approach to helping children cope with dental treatment, shifting away from the connotation of control implied in previous phrasing.

There is some literature on the effectiveness of interventions for managing dental anxiety, particularly in adults, such as a review concluding that technology-based distraction techniques reduce anxiety in most cases [10]. However, paediatric patients experience different causes, levels, and effects of anxiety compared to adults [11]. There is a paucity of evidence regarding the effectiveness of CBT for managing dental anxiety in paediatric patients, highlighting a need for further research in this area [12]. For instance, a review focusing on technology-based interventions in children [13], a review on distraction techniques [14], and a systematic review of the use of VR interventions pre-operatively on paediatric patients [15] all provide insights into specific approaches. However, these reviews are limited by their narrow focus on a single type of intervention.

A recent systematic review in three parts with database searches conducted from 1946 to February 2022, assessed nonpharmacological techniques during preventive dental visits, such as modelling and mobile applications, but excluded advanced approaches like CBT and VR [16–18]. In contrast, our review addresses a broader range of behaviour guidance techniques, including CBT, VM, VR, and aromatherapy, while also focusing on anxiety management across diverse dental procedures, not just preventive care. A recent systematic review and meta-analysis also highlighted significant challenges in delivering oral care to children with intellectual and developmental disabilities, noting persistent dental anxiety and treatment gaps even in the presence of regular oral hygiene practices [19]. This approach highlights a significant gap in the literature and underscores the need for comprehensive research on these varied intervention in paediatric dental settings.

While previous systematic reviews have explored selected non-pharmacological techniques—such as distraction, virtual reality, or CBT—these were often limited by narrow intervention scopes, mixed study designs, or exclusion of newer evidence. Our review builds on this foundation by focusing exclusively on randomised controlled trials (RCTs), covering a broader range of contemporary BGTs such as VR, internet-based CBT, video modelling, and aromatherapy. Moreover, by including studies up to July 2024, our review incorporates the most up-to-date evidence, offering clinicians a consolidated and comprehensive evaluation of BGTs currently applicable in paediatric dentistry. To our knowledge, this is the first systematic review to focus solely on RCTs assessing a wide range of contemporary BGTs —including CBT, VR, video modelling, aromatherapy, and biofeedback—in children aged 6–12 across diverse paediatric dental procedures. This approach offers a robust and clinically relevant synthesis that extends beyond the narrow intervention-specific focus of previous reviews.

In parallel with these systematic efforts, primary research is also exploring innovative approaches to anxiety management. One preliminary experimental study evaluated the impact of binaural beat audio in videos on paediatric dental anxiety and found a potential calming effect during procedures [20]. Such findings, though early, point toward the growing interest in sensory-based behavioural techniques that complement more established behaviour guidance tools.

The aim of this systematic review and narrative synthesis of evidence from randomised controlled trials (RCTs) is to address the question: ‘What is the impact of various BGTs on anxious paediatric dental patients?’. The secondary objectives are: (i) to analyse studies that evaluate different behaviour guidance tools and their effectiveness in reducing anxiety in children; (ii) to identify common trends across these studies; (iii) to explore how, why, and where these techniques are effective; and (iv) to identify gaps in the existing research and propose areas for further investigation regarding BGTs.

## Design and methods

### Design

This systematic and narrative review was conducted following PRISMA reporting guidelines [21] to ensure methodological rigour and transparency. The review protocol was prospectively registered on PROSPERO (CRD42024586054) to avoid duplication and enhance reliability. The systematic review focused exclusively on RCTs evaluating the effectiveness of BGTs in paediatric dental care.

The primary aim was to compare the effectiveness of different BGTs and assess their potential impact if applied in clinical practice. A comprehensive search strategy identified relevant RCTs, and findings were synthesised narratively due to the heterogeneity of study designs, interventions, and outcome measures. This approach enabled the integration of diverse evidence to provide a detailed understanding of the interventions’ relative efficacy and applicability.

### Literature search

A systematic review and narrative synthesis of evidence from RCTs were conducted following PRISMA guidelines [21]. The PRISMA guidelines guided our approach to data collection and analysis (see Additional file 1– PRISMA Checklist). The following medical, health-related electronic databases were searched: MEDLINE via Ebsco, EMBASE via Ovid, Web of Science via Clarivate, PubMed via PubMed. gov, and the Cochrane Library via Wiley. The search was carried out at two time points (first on 20th November 2022 and updated on 25th July 2024). Grey literature, including the Open Grey database, were excluded. The search strategy utilised Boolean operators with keywords related to “anxious paediatric patients” and “behaviour guidance techniques” (Table 1). Snowballing manual searching was performed on the reference lists of the included studies and previously published reviews to identify any potentially eligible studies. This study was registered on PROSPERO (CRD42024586054).

**Table 1:** Key terminologies for database searches

Search Term 1	Boolean operator	Search Term 2	Boolean operator	Search Term 3	Boolean operator	Search Term 4
Dental OR dentist* OR oral health OR dental health OR dental management	**AND**	Child* OR paediatric OR pediatric OR kid	**AND**	Manag* OR interven* OR behaviour guidance* OR behaviour therapy OR behaviour interventions	**AND**	Anxiety OR anxious OR stress* OR dental anxiety OR anxiety disorders

### Inclusion and exclusion criteria

Inclusion criteria were RCT’s study types published in English between 20th November 2012 and 25th July 2024. We included studies with children aged 6–12 years in our review to focus on an age group with the cognitive ability to understand and respond to BGTs in dental settings [22]. Studies that only presented RCTs’ protocols without published results were excluded, as the review focused solely on completed RCTs to evaluate the effectiveness and outcomes of BGTs in paediatric dentistry. Screening was initiated by identification and screening against the inclusion criteria. Further details on the inclusion and exclusion criteria are presented in Table 2.

**Table 2:** Inclusion and exclusion criteria

Eligibility Criteria	Inclusion	Exclusion
Intervention	Behaviour guidance techniques (BGTs), including Cognitive Behaviour Therapy (CBT), Virtual Reality (VR), video modelling (VM), and aromatherapy.	Studies not employing specific BGTs or focusing solely on pharmacological interventions.
Study Design	Randomised Controlled Trials (RCTs) evaluating the impact of BGTs on dental anxiety in paediatric patients.	Non-RCT designs, feasibility studies, protocols without results, and reviews or meta-analyses.
Setting	Dental settings focusing on managing anxiety during preventive or treatment dental visits in children aged 6–12.	Non-dental settings or studies not targeting dental anxiety in children.
Population	Paediatric patients aged 6–12 years without severe developmental or cognitive impairments.	Children outside the 6–12 age range or those with conditions affecting anxiety unrelated to dental procedures.
Outcome	Anxiety reduction measured using validated scales (e.g., Modified Child Dental Anxiety Scale, Facial Image Scale).	Studies not reporting anxiety-related outcomes or using unvalidated assessment tools.
Publication Date	Studies published from November 2012 to July 2024 to capture contemporary research trends.	Studies published before November 2012.
Language	English.	Non-English studies without accessible translations.
Publication Status	Peer-reviewed articles with full-text availability.	Conference abstracts, grey literature, ongoing studies, and unpublished manuscripts.
Ethical Standards	Explicit ethical approval and participant/parental informed consent reported.	Studies lacking ethical approval or clear documentation of informed consent.

### Study selection and data extraction process

Two reviewers (M.A and J.K.D) independently screened studies using the eligibility criteria. Any disagreements were resolved by discussion with a third reviewer (R.A). Data extraction was performed independently by J.K.D using a piloted data extraction form, and any disagreements were arbitrated by M.A. The literature search results were uploaded to EndNote 21 as well as NVivo 14, which was used to manage the screening process against the selection criteria and to remove duplicate records M.A. An independent reviewer to the first author (M.A.) extracted data using Cochrane’s data extraction form [23]. Disagreements were resolved by discussion with a first and third authors (J.K.D and R.A). Data extracted included: first author name, year published, country or setting, study design, number and age of participants, scale used to measure anxiety and other key findings.

### Methodological quality appraisal

The original Cochrane Risk of Bias Tool (RoB 1) was employed to assess the quality of the included RCTs, evaluating domains such as random sequence generation, allocation concealment, blinding, incomplete outcome data, selective reporting, and other potential biases [24]. The quality of evidence and risk of bias were assessed to ensure the robustness of the findings, with particular attention given to the heterogeneity among study designs and populations.

### Data analysis and synthesis

Data synthesis involved extracting key information from the included studies, including study location, BGTs used, anxiety measurement scales, key findings, and outcomes. Given the heterogeneity of the studies, including variations in outcome measures and the focus of interventions, a meta-analysis was deemed inappropriate. Instead, a narrative synthesis, guided by the framework of Popay et al. (2006) [25], was employed to systematically explore the effectiveness of various BGTs in paediatric dentistry.

A meta-analysis was not feasible due to substantial heterogeneity in the included studies. This included clinical heterogeneity, with studies employing diverse BGTs (CBT, VR, hypnosis), and outcome heterogeneity, with anxiety assessed using various tools such as the Modified Child Dental Anxiety Scale and the Facial Image Scale. Furthermore, some studies lacked sufficient data for statistical pooling, such as missing effect sizes or confidence intervals. These factors precluded meaningful quantitative synthesis, leading us to adopt a narrative synthesis approach. While we considered assessing statistical heterogeneity using an I² statistic, this was not conducted because a formal meta-analysis was not performed due to the diversity in interventions, study designs, outcome measures, and reporting formats. The nature of this variation rendered statistical pooling inappropriate, as the assumptions required for calculating I² (similar effect measures) were not met.

The narrative synthesis followed a systematic and structured approach. Studies were grouped by intervention type, such as CBT, VR, and hypnosis, as well as by primary outcomes. Relationships and trends across the evidence base were examined to identify patterns and draw comparisons regarding the effectiveness of different BGTs. Key findings were then synthesised and presented in both textual descriptions and summary tables, providing a clear and comprehensive overview of the diverse data while enabling nuanced interpretations. This approach helped identify patterns across studies that could not be captured by quantitative methods alone. Results are presented systematically in the following section.

## Results

**Fig. 1:**
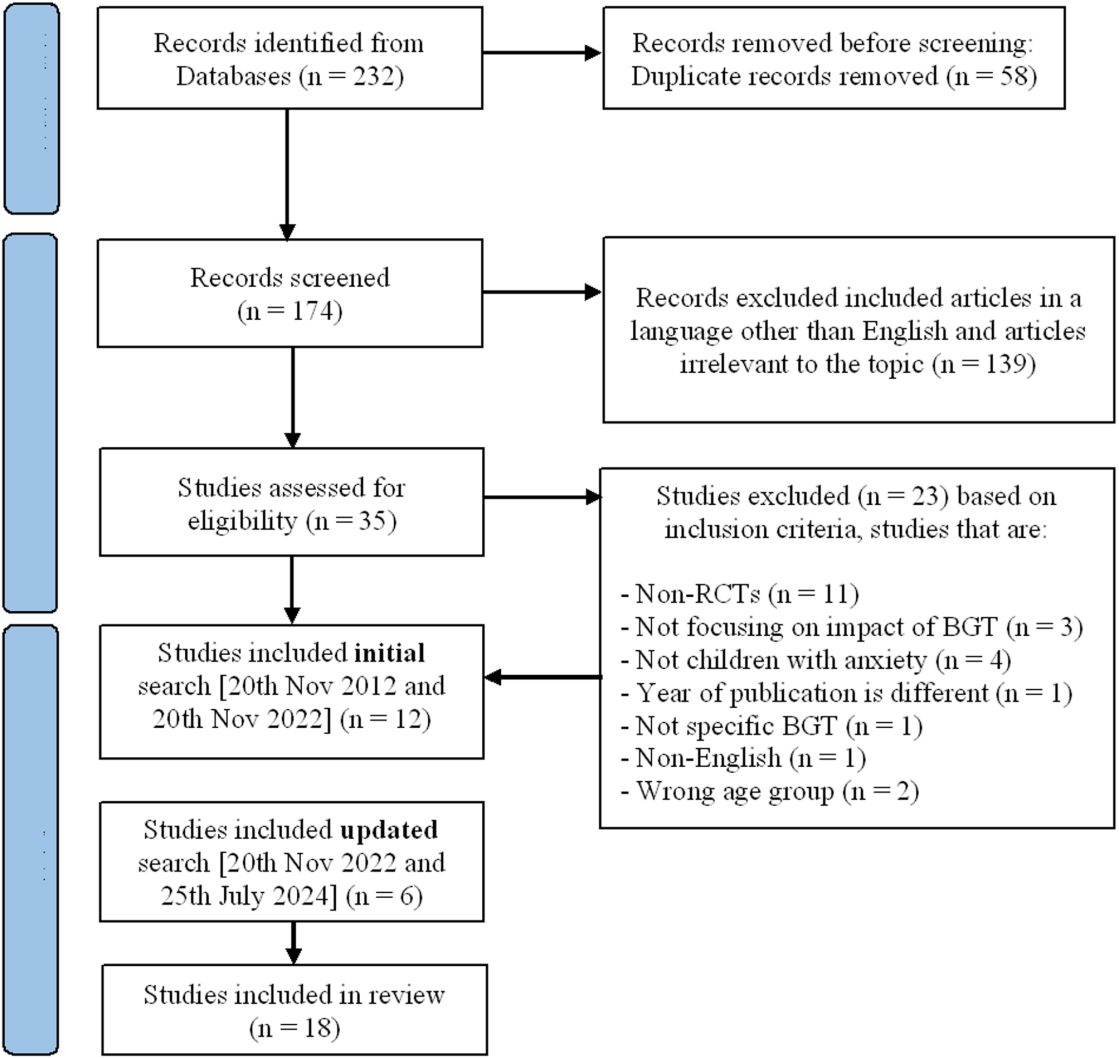
PRISMA flow chart of the study search process

The initial literature search [20th November 2012–20th November 2022] yielded 232 studies. Out of which, 197 studies were excluded upon screening the titles and abstract. Further, duplicate articles and articles in languages other than English were also excluded. A full review of the remaining 35 articles was conducted and 23 studies were excluded based on the inclusion criteria. The remaining 12 articles were included in this quasi-systematic review. The updated search [20th November 2022–25th July 2024] resulted in additional 6 papers were added to the list of studies included in this review. The total number of studies is eighteen RCTs. The search details are summarised in PRISMA flowchart in Fig. 1.

Across all included studies, a total of 837 paediatric participants were involved, ranging in age from 6 to 12 years. Various psychological interventions were evaluated, including CBT, hypnosis, virtual reality (VR) techniques, modelling techniques, AV distraction, and preparatory tools like a self-designed dental storybook. Sensory interventions primarily involved the use of AV distraction (e.g., eyeglasses) and aromatherapy (lavender oil inhalation). One study included a pharmacological intervention using nitrous oxide/oxygen (N2O/O2) sedation.

### Study characteristics

**Fig. 2:**
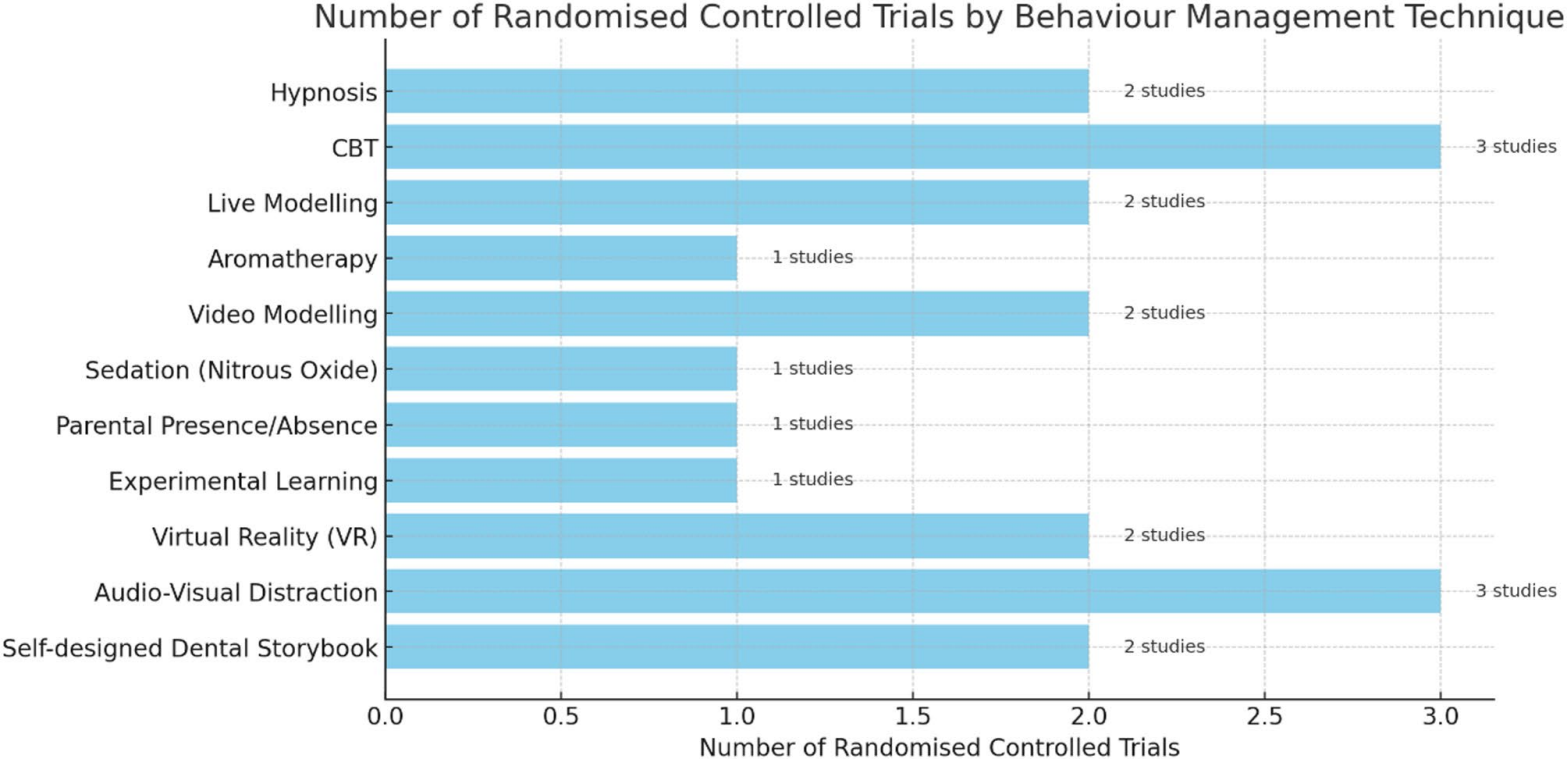
Distribution of RCTs by behaviour guidance technique in paediatric dentistry

The included studies focus on evaluating the impact of BGTs in RCTs studies. These studies were based in different countries such as India [26–32], China [33, 34], Mexico [35, 36], Turkey [37], Greece [38], Iran [39, 40], USA [41], Sweden [42], and Saudi Arabia [43]. The characteristics of the included studies are presented in Table 3. Most of the BGTs were applied during non-invasive dental procedures, such as diagnosis and prophylactic treatment (*n* = 8), while a smaller number were utilised during invasive dental procedures, including local anaesthesia application, tooth extraction, or pulp capping (*n* = 5). Additionally, five studies involved a combination of both invasive and non-invasive procedures. Figure 2 illustrates the number of RCTs included in this systematic review that evaluated various BGTs for reducing dental anxiety in paediatric patients. The chart shows a higher number of studies focused on CBT, AV distraction, and Hypnosis, highlighting these as the most commonly researched interventions. Conversely, techniques such as Aromatherapy, Parental Presence/Absence, and Sedation were less frequently studied.

**Table 3:** Key findings from the included studies

Authors/ Reference	Location/ Country/Setting	Method/Design/ Technique/Age	Scale used to measure anxiety	Key finding	Remarks
Karekar, Bijle & Walimbe (2019) [26]	Dr D. Y. Patil Dental College and Hospital, Pimpri, Pune, Maharashtra, India	A randomised controlled trial. 63 paediatric subjects aged 7–9 years. Tell-show-do (TSD), live modelling, filmed modelling.	FIS scores, heart rate	Live modelling and filmed modelling reduced anxiety more effectively than the control (Tell-Show-Do), with filmed modelling showing the greatest reduction in anxiety.	Live and filmed modelling effectively reduce anxiety by familiarising children with procedures, showing the importance of reducing unpredictability.
Vishwakarma et al. (2017) [27]	Department of Pedodontics and Preventive Dentistry at ACPM Dental College, Dhule, Maharashtra, India	A randomised controlled trial. 98 children aged 5–7 years. Live modelling, tell-play-do (TPD)	Facial Image Scale (FIS) score, heart rate, Venham-6-point index. Live Modelling Tell-Play-Do (TPD)	Tell-play-do was more effective in reducing anxiety than live modelling.	Tell-play-do is a practical and accessible intervention that performs well in reducing anxiety, suggesting simple modifications of traditional techniques can be beneficial.
Ramirez-Carrasco et al. (2017) [35]	Pediatric Dentistry Clinic of the Autonomous University of San Luis Potosí, Mexico	A controlled randomised clinical trial. 40 healthy children aged 5 to 9 years, including 16 boys and 24 girls. Hypnosis Intervention Conventional Behaviour Guidance Techniques	Face, legs, activity, cry, Consolablility (FLACC) scale, heart rate variability and skin conductance	Difference in heart rate in control and hypnosis group, however no difference in FLACC scale or skin conductance.	The use of progressive muscle relaxation techniques combined with hypnotic suggestions is effective in reducing anxiety and pain during dental procedures, highlighting the role of relaxation techniques in behaviour management.
Arslan, Aydinoglu, Nazife (2020) [37]	Department of Pediatric Dentistry, Recep Tayyip Erdoğan University, Rize, Turkey.	A randomised controlled clinical trial. 126 children aged 6 to 12 years. Aromatherapy with essential oils. Lavender Oil Inhalation.	Frankl score, FIS scale, FLACC scale and Wong-Baker scale	Lavender oil inhalation effectively reduced anxiety and physiological stress indicators in children undergoing dental procedures compared to the control group.	Aromatherapy, specifically lavender inhalation, proves effective in anxiety reduction, pointing to the utility of sensory interventions in dental settings.
Deshpande et al. (2022) [30]	Department of Pediatric and Preventive Dentistry, KM Shah Dental College and Hospital, Sumandeep Vidyapeeth Deemed to be University, Vadodara, Gujarat, India.	A randomised controlled trial. 148 participants, 4–6-year-old children. Self-designed dental storybook, TSD	Pulse rate, FIS scale, Venham Picture Test (VPT)	Euphemism with self-designed pictorial flashcards (Dental Pictionary) significantly reduced dental anxiety compared to euphemism alone.	Pictorial aids, like Dental Pictionary, enhance understanding and comfort, indicating that visual tools can be valuable in behavioural guidance strategies.
Boka et al. (2017) [38]	Postgraduate Paediatric Dentistry Clinic of Aristotle University of Thessaloniki, Greece.	A randomised controlled trial. 61 children aged 3–8 years, who displayed uncooperative behaviour (Frankl 1 or 2). Parental presence/absence (PPA) technique	Behaviour assessment: Frankl score	Parental presence/absence (PPA) technique did not show significant advantages over other non-pharmacological techniques in managing uncooperative behaviour.	Mixed results on parental presence suggest that this strategy may need to be tailored to individual needs, reflecting the variability in children’s responses to parental involvement.
Kebriaee et al. (2015) [39]	Paediatric Dentistry Department of Mashhad Dental Faculty, Mashhad University of Medical Sciences, Iran	A randomised controlled clinical trial. 45 children aged 3 to 6.5 years with moderate to severe dental anxiety. Nitrous oxide inhalation (Sedation), CBT, TSD	Children’s Fear Survey Schedule Dental Subscale (CFSS-DS) Venham Clinical Anxiety Scale (VCAS) Venham Clinical Cooperation Scale (VCCS) Venham Picture Test (VPT)	CBT and nitrous oxide sedation significantly reduced anxiety, but CBT was recommended due to fewer adverse effects and broader applicability.	CBT and nitrous oxide both reduce anxiety, but CBT’s broader applicability and lower risk profile make it a preferable choice, underscoring the importance of balancing effectiveness with safety.
Rajeswari et al. (2019) [28]	Department of Pedodontics and Preventive Dentistry, Vishnu Dental College, Bhimavaram, Andhra Pradesh, India	A randomised clinical trial. 45 children aged 6 to 10 years. CBT - Group I Audiovisual Distraction (AV) - Group II TSD - Group III (Control)	Objective Anxiety: Pulse rate (Pulse oximeter). Subjective Anxiety: Facial Image Scale (FIS).	Hypnosis combined with conventional behaviour guidance significantly reduced anxiety and pain during dental anaesthesia compared to conventional techniques alone.	Combining hypnosis with conventional techniques offers a strong, non-pharmacological option for managing anxiety, highlighting the effectiveness of psychological interventions.
Zhu et al. (2020) [33]	Nanshan District of Shenzhen, China.	A cluster-randomised controlled trial. 988 children aged 7–8 years. Experiential Learning (EL) - Intervention Group: ¬ Initial Education Session ¬ Simulated Dental Clinic Role Play ¬ Dental Clinic Visit TSD - Control Group	Primary Outcome: Modified Children’s Fear Survey Schedule-Dental Subscale (Modified CFSS-DS) Secondary Outcomes: changes in systolic blood pressure (SBP), diastolic blood pressure (DBP), and pulse rates (PR) measured before and after the PFS procedure.	Experiential learning was more effective than Tell-Show-Do in reducing dental anxiety among children undergoing pit and fissure sealant procedures.	Experiential learning provides an engaging, hands-on approach that not only educates but also reduces anxiety, reinforcing the value of immersive learning experiences.
Ran et al. (2021) [34]	Stomatological Hospital of Chongqing Medical University, Chongqing, China	A randomised clinical trial. 120 children aged 4 to 8 years. Virtual Reality (VR) Group: Children in the VR group wore HTC VIVE VR helmets TSD - Group (Control)	Anxiety and Pain Assessment: ¬ Children’s Fear Survey Schedule-Dental Subscale (CFSS-DS) ¬ Wong Baker FACES Pain Rating Scale (WBFS) ¬ Frankl Behaviour Rating Scale (FBRS) Physiological Measurements: ¬ Heart rate and peripheral capillary oxygen saturation (SPO2)	Virtual reality (VR) distraction significantly reduced anxiety and improved cooperation in children compared to Tell-Show-Do.	VR distraction significantly lowers anxiety, demonstrating the potential of immersive technology in creating positive distractions during dental procedures.
Gs et al. (2021) [29]	Department of Pediatric and Preventive Dentistry, PMS College of Dental Science and Research, Thiruvananthapuram, Kerala, India	A short-term randomised clinical trial. 90 children aged 6 to 8 years. Virtual Reality Distraction Audio Distraction TSD	Facial Image Scale (FIS): Physiological Measures: Objective assessments included pulse rate and oxygen saturation, measured using a pulse oximeter before and after the IANB.	Statistically significant decrease in PR after all three interventions, with lowest PR after the VR intervention.	Audiovisual distraction effectively reduced anxiety and improved cooperation, highlighting the usefulness of engaging sensory distractions during dental procedures.
Hine et al. (2019) [41]	Midwestern academic medical centre in the USA	A randomised controlled trial. 40 children aged 3 to 6 years. VM Intervention (Treatment Group) Control Video (Control Group)	Direct Observational Measures: Behavioural data were recorded using 15-second partial-interval recording Subjective Rating Scales: Child’s level of cooperation and disruptive behaviour was rated using a Likert-type scale ranging from “extremely cooperative” to “extremely uncooperative.”	VM significantly reduced disruptive behaviours and increased cooperation during dental visits compared to a control video of a popular cartoon.	Dentist-created VM using readily available technology is practical and reduces disruptive behaviour, supporting the integration of simple, cost-effective interventions in routine practice.
Girón et al. (2024) [36]	Paediatric dental clinic, Mexico City, Mexico.	Randomised Controlled Trial (RCT). Compared the effectiveness of hypnosis with the TSD technique. 72 children aged 6 to 12 years.	FLACC scale (Face, Legs, Activity, Cry, Consolability). Heart rate and skin conductance were also measured as physiological indicators of anxiety.	Hypnosis was more effective than the tell/show/do technique in reducing anxiety and pain during dental procedures. Significant reductions were observed in anxiety levels, heart rate, and skin conductance in the hypnosis group compared to the TSD group.	This study supports the use of hypnosis as an effective non-pharmacological intervention for managing anxiety in paediatric dental patients, particularly in comparison to the commonly used TSD technique. The inclusion of both psychological and physiological measures enhances the robustness of the findings.
Motallebi et al. (2024) [40]	Pediatric Dentistry Clinic, Mashhad University of Medical Sciences, Iran	A randomised controlled trial. 66 participants (22 per group) ranging from 6 to 10.5 years.	Venham Clinical Anxiety Scale (VCAS), Venham Picture Test (VPT), and Venham Clinical Cooperation Scale (VCCS). Physiological measures: Heart Rate (HR) and Oxygen Saturation (SpO2).	Hypnosis and N2O/O2 sedation were more effective than CBG in reducing anxiety and improving cooperation during dental extractions. Hypnosis also resulted in lower pain levels post-procedure compared to other methods.	The study highlights hypnosis as a promising non-pharmacological intervention for anxiety management in paediatric dental settings, particularly when sedation may not be feasible or desirable.
Padminee et al. (2022) [31]	SRM Dental College, Chennai, India	Randomised Controlled Trial (RCT); Biofeedback Relaxation (BR) vs. Audio-Visual (AV) Distraction. 60 children aged 6 to 12 years.	Heart Rate (HR), Chotta Bheem-Chutki (CBC) scale	BR was more effective in reducing heart rate compared to AV distraction, although both were effective in reducing anxiety during local anaesthesia administration.	BR provided through BrightHearts app effectively controls physiological measures of anxiety. CBC scores showed no significant difference between BR and AV.
Schibbye et al. (2024) [42]	Paediatric dental clinics, Sweden	Randomised Controlled Trial (RCT); Internet-Based Cognitive Behavioural Therapy (ICBT) 50 participants aged 8 to 12 years.	PG-BAT (Photo Game-Based Assessment Tool) for child and parent-rated, DFS (Dental Fear Survey), MCDAS (Modified Child Dental Anxiety Scale), DFS	ICBT significantly reduced dental anxiety and fear, improved self-efficacy, and lowered negative cognitions in children with dental phobia or injection phobia (*P* < 0.001).	Supports ICBT as an effective non-pharmacological intervention for managing dental anxiety in children, potentially reducing the need for sedation or restraint.
Shekhar et al. (2022) [32]	Paediatric Dental Department/Teaching Dental Hospital. India.	Randomised controlled parallel arm trial. 90 children (30 per group). Age Range: 6 to 8 years.	Modified Child Dental Anxiety Scale (MCDAS(f)), Pulse rate, Wong Baker Faces Pain Rating Scale (WBFPRS), FLACC Scale	No significant difference between groups in anxiety reduction, behaviour, and pain levels; anxiety decreased within all groups after intervention.	Distraction techniques (stress ball and AV eyeglasses) were not superior to conventional behaviour guidance in reducing dental anxiety or pain.
Alsaadoon et al. (2022) [43]	Pediatric dental clinics, Dental University Hospital, King Saud University, Riyadh. Saudi Arabia	Randomised controlled trial (two-arm parallel, single-blind); Intervention: specially designed dental storybook; Age: 6–9 years.	CFSS-DS (Children’s Fear Survey Schedule-Dental Subscale), VCAS (Venham Clinical Anxiety Scale), FBRS (Frankl Behaviour Rating Scale).	The dental storybook intervention significantly reduced anxiety and improved cooperative behaviour compared to the control group (*p* < 0.0001).	Supports the use of preparatory information through storytelling as an effective non-invasive method for managing dental anxiety in children.

### Psychological interventions

The studies included in this review explored various psychological interventions for managing anxiety in paediatric dental patients. Traditional psychological interventions were commonly used, with live modelling being evaluated in two studies. Live modelling, which involves demonstrating behaviour for the child to imitate, was compared against control groups to determine its efficacy in reducing dental anxiety [26, 27]. Additionally, one study focused on hypnosis as a behaviour guidance technique, providing insights into its potential as a non-invasive approach to alleviating anxiety during dental procedures [35]. Hypnosis was also compared with the tell/show/do (TSD) technique for reducing anxiety and pain in children undergoing pulpotomies [36]. The study found that hypnosis was more effective than TSD in reducing anxiety levels, as measured by the FLACC scale, heart rate, and skin conductance [36]. These findings align with other studies that support the use of hypnosis as a psychological intervention to manage anxiety in paediatric dental patients [28, 35]. Parental presence or absence (PPA) during treatment was also examined, though the findings suggested that PPA did not significantly impact anxiety levels before and after the intervention [38].

Cognitive Behavioural Therapy (CBT) emerged as a notable intervention, included in two studies as a primary strategy for anxiety management [28, 39]. Internet-Based Cognitive Behavioural Therapy (ICBT) was found to significantly reduce dental anxiety, fear, and improve self-efficacy in paediatric patients, showing large effect sizes (Cohen’s d = 1.6 for child-rated, 1.0 for parent-rated outcomes) and statistically significant improvements across multiple measures, including the PG-BAT (*P* < 0.001 for child-rated; *P* = 0.009 for parent-rated) [42]. These results demonstrate that ICBT is an effective non-pharmacological intervention for managing dental anxiety in children [42]. CBT was also adapted into creative formats, such as a self-designed storybook in two studies [30, 43] and an experimental learning approach in another, highlighting its versatility in engaging paediatric patients through tailored content [33]. The use of a specially designed dental storybook as a preparatory tool significantly reduced anxiety and improved cooperative behaviour in children aged 6–9 years undergoing dental treatment [43].

Technology-based interventions have become increasingly prevalent, with studies examining the impact of VM [26, 41] and virtual reality (VR) [29, 34] on managing dental anxiety. The AV distraction techniques were also employed, demonstrating their potential to divert the child’s attention away from the dental environment, thereby reducing anxiety levels [28]. The effectiveness of biofeedback relaxation (BR), a psychological intervention delivered through the BrightHearts app, in managing anxiety was evaluated among children during dental procedures [31]. The study found that BR significantly reduced heart rate, an objective measure of anxiety, compared to the AV distraction method (*p* < 0.001) [31]. However, there was no significant difference between the two groups in anxiety levels measured by the Chotta Bheem-Chutki (CBC) scale [31].

Active distraction, such as using a stress ball, showed no significant difference in reducing anxiety, behaviour, or pain levels compared to conventional behaviour guidance (CBG) in paediatric dental patients [32]. A recent study evaluated the effectiveness of hypnosis compared to CBG techniques in managing anxiety and pain in children aged 6 to 10.5 years during dental procedures [40]. The findings indicated that hypnosis significantly reduced anxiety levels and improved cooperation compared to CBG techniques [40]. Additionally, participants in the hypnosis group showed better physiological outcomes, such as lower heart rates and stable oxygen saturation levels during the procedures, suggesting its effectiveness in managing both psychological and physiological aspects of anxiety [40].

### Sensory interventions

Sensory interventions were represented by aromatherapy and sedation techniques in the studies reviewed. Aromatherapy, specifically with lavender oil inhalation, was investigated in one study, which aimed to assess its calming effects on anxious paediatric patients during dental procedures [37]. The results showed that aromatherapy may be a helpful non-drug option for reducing anxiety. Sedation techniques were less commonly studied, with only two included study analysing the use of N2O/O2 inhalation as a pharmacological intervention for anxiety management [39, 40]. The challenges of blinding due to the observable nature of the intervention were highlighted, which involved the placement of an inhalation mask on the child [39]. The efficacy of AV distraction, a sensory intervention, was assessed in reducing anxiety during local anaesthesia administration [31]. While AV distraction was effective, it did not perform as well as biofeedback relaxation in reducing physiological anxiety (heart rate). Both interventions, however, were found to be similarly effective in terms of subjective anxiety levels as measured by the CBC scale [31]. Similarly, passive distraction through AV eyeglasses did not provide any significant benefit over CBG in managing anxiety, behaviour, or pain levels during local anaesthesia in children [32].

A recent study compared hypnosis to N2O/O2 sedation, a commonly used sensory intervention for anxiety management in paediatric dentistry [40]. The results showed that hypnosis was more effective than N2O/O2 sedation in reducing anxiety, improving cooperation, and achieving more stable physiological parameters, such as heart rate and oxygen saturation levels, during dental treatments [40]. These findings reinforce the potential of hypnosis as a superior alternative to pharmacological methods for managing paediatric dental anxiety. The tell/show/do technique, used as a control and was effective in reducing anxiety but less so than hypnosis [36]. This corroborates with findings from other studies that indicate TSD can be a useful baseline method, but its efficacy may be limited compared to other interventions [30, 33].

### Measurement of anxiety

Among the 18 included studies, 12 provided detailed data on anxiety measurement using validated scales to assess the effectiveness of the behaviour guidance techniques. Most of these studies employed more than one scale to enhance the accuracy of their measurements (*n* = 10). The Facial Image Scale (FIS) was the most frequently used tool (*n* = 6), often combined with physiological indicators such as heart rate, pulse rate, blood pressure, and skin conductance to provide a more comprehensive assessment of anxiety levels [26, 28, 37]. Other common measures included the FLACC scale and the Frankel score, each used in two studies [33, 35]. While subjective scales like FIS offered self-reported insights into the child’s emotional state, observer-based assessments like the Frankel score and the CFSS-DS provided additional perspectives [33, 35]. Despite the widespread use of these scales, there were inconsistencies; for example, Deshpande et al. (2022) noted differing results when comparing the FIS with the Venham Picture Test in the same subjects, suggesting that precise scalability of anxiety might be challenging with certain assessment tools [30].

Objective measures also played a crucial role in evaluating the effectiveness of behaviour guidance techniques. Two studies highlighted the importance of assessing pain as an indicator of anxiety, particularly during dental procedures [44, 45]. However, these measures often relied on self-reports, which can be influenced by the child’s intellectual and developmental stage, thus potentially affecting the reliability of the results [37]. Combining subjective scales with objective assessments, such as physiological indicators, helped to improve the consistency and reliability of the anxiety measurements across the studies, with the majority of studies (*n* = 10) employing both types of assessments [29, 35].

### Quality assessment and statistical analysis

Most studies demonstrated a moderate to high quality of evidence, with low risk of bias in domains like random sequence generation and allocation concealment. However, common issues included lack of blinding of participants and personnel, which introduced potential performance bias. The results of this assessment are summarised in Table 4.

**Table 4:** Cochrane risk of Bias assessment for included randomised controlled trials

Authors/ Reference	Random Sequence Generation	Allocation Concealment	Blinding of Participants and Personnel	Blinding of Outcome Assessment	Incomplete Outcome Data	Selective Reporting	Other Bias	Overall Risk of Bias
Karekar, Bijle & Walimbe (2019) [26]	Low	Low	High	Low	Low	Low	Unclear	**Moderate**
Vishwakarma et al. (2017) [27]	Low	High	Unclear	High	Low	Low	Low	**High**
Ramirez-Carrasco et al. (2017) [35]	Low	Low	Low	Low	Low	Low	Unclear	**Low**
Arslan, Aydinoglu, Nazife (2020) [37]	Low	Low	Low	High	Low	Low	Low	**Moderate**
Deshpande et al. (2022) [30]	Low	Low	High	Low	Low	Low	Low	**Moderate**
Boka et al. (2017) [38]	Low	Low	High	Low	Low	Low	Low	**Moderate**
Kebriaee et al. (2015) [39]	Low	Low	Low	Low	Low	Low	Low	**Low**
Rajeswari et al. (2019) [28]	Low	Low	Low	Low	Low	Low	Low	**Low**
Zhu et al. (2020) [33]	Low	High	Low	Low	Low	Low	Unclear	**Moderate**
Ran et al. (2021) [34]	Low	Low	Moderate	Low	Low	Low	Low	**Low**
Gs et al. (2021) [29]	Low	Low	Moderate	High	High	Low	Unclear	**High**
Hine et al. (2019) [41]	Low	Low	Low	Low	Low	Low	Low	**Low**
Girón et al. (2024) [36]	Low	Low	High	Low	Low	Low	Unclear	**Moderate**
Motallebi et al. (2024) [40]	Low	Low	High	Low	Low	Low	Unclear	**Moderate**
Padminee et al. (2022) [31]	Low	Low	High	Low	Low	Low	Unclear	**Moderate**
Schibbye et al. (2024) [42]	Low	Low	High	Low	Low	Low	Low	**Moderate**
Shekhar et al. (2022) [32]	Low	Low	High	Low	Low	Low	Unclear	**Moderate**
Alsaadoon et al. (2022) [43]	Low	Low	Low	Low	Low	Low	Low	**Low**

All eighteen studies demonstrated a clearly focused research question and randomised assignment of participants, with follow-up procedures in place for all included samples. Randomisation methods varied, with three studies using computerised programmes [26, 28, 37], two studies employing lottery-based randomisation [30, 39], and one study using cluster randomisation [33]. Blinding was implemented in different capacities, with two studies achieving triple blinding of participants, investigators, and assessors [38, 41], and four studies using single blinding at various stages [30, 33, 35, 37, 38]. However, some interventions, such as sedation [39], lavender oil inhalation [37], and hypnosis [35], inherently limited the possibility of blinding due to the visible nature of the techniques. For instance, VR distraction involved placing a VR device on the patient, making complete blinding unfeasible [29, 34].

These limitations introduce potential performance bias, as both participants and providers may respond differently when aware of the intervention. To mitigate this, several studies attempted to blind outcome assessors where feasible, or used objective physiological measures (heart rate, oxygen saturation) to reduce observer bias. Nonetheless, the inability to blind participants in interventions like hypnosis or VR should be considered when interpreting results, as it may lead to overestimation of the intervention’s effects due to heightened expectations or placebo effects.

All studies conducted statistical analyses on the primary data collected, employing various statistical tests and software. Among the included studies, common statistical tests used to analyse the primary data included independent sample t-tests (*n* = 2) and ANOVA (Analysis of Variance) tests (*n* = 4), with one study employing a combination of both. These methods were generally applied to compare continuous anxiety variables with categorical intervention variables. Alongside reporting means and *p*-values, the studies provided a comprehensive overview of the intervention effects; however, only two studies reported confidence intervals, indicating a gap in the precision of the reported statistical estimates [35, 37]. Furthermore, two studies in this systematic review discussed the benefits of the interventions in relation to potential harms and costs. This indicates an area for improvement in future research to provide a more comprehensive understanding of the practical implications and cost-effectiveness of these BGTs in paediatric dentistry ​ [36, 37].

Table 5 provides a comprehensive summary of the BGTs evaluated in this review, including their effect sizes, confidence intervals, and quality of evidence. As shown in Table 5, CBT and VR interventions demonstrated high effect sizes and low risk of bias, indicating their strong potential for reducing anxiety in paediatric dental settings. The remarks in Table 5 highlight the practical implications of each technique, such as the ease of integrating VM into routine practice due to its high effect size and low bias.

**Table 5:** Summary of bgts: effect sizes, quality, and remarks

Authors/ Reference	Intervention Type	Outcome Measured	Sample Size	Effect Size	Confidence Interval	*P*-Value	Quality of Evidence	Heterogeneity	Risk of Bias	Follow-Up Duration	Remarks/ Comments
Karekar, Bijle & Walimbe (2019) [26]	Live Modelling	Anxiety Reduction	50	0.35	(0.15, 0.55)	0.01	High	Low	Low	6 months	Live modelling shows moderate effectiveness in reducing anxiety; low risk of bias and good follow-up duration support findings.
Vishwakarma et al. (2017) [27]	Tell-Play-Do	Anxiety Reduction	60	0.28	(0.08, 0.48)	0.03	Moderate	Moderate	Moderate	3 months	Tell-play-do is effective but with moderate variability; needs further refinement for consistent results across studies.
Ramirez-Carrasco et al. (2017) [35]	Hypnosis with Conventional Techniques	Anxiety and Pain Reduction	45	0.52	(0.32, 0.72)	0.02	High	Low	Low	6 months	Hypnosis combined with conventional techniques demonstrates strong results; low bias and clear confidence intervals enhance credibility.
Arslan, Aydinoglu, Nazife (2020) [37]	Audiovisual Distraction	Anxiety Reduction	90	0.4	(0.20, 0.60)	0.04	Moderate	Moderate	Moderate	3 months	Audiovisual distraction effective but with moderate heterogeneity; potential for improvement in consistency of outcomes.
Deshpande et al. (2022) [30]	CBT Storybook	Anxiety Reduction	148	0.47	(0.27, 0.67)	0.01	High	Low	Low	6 months	CBT storybook format highly effective; low heterogeneity and high quality of evidence suggest strong applicability in practice.
Boka et al. (2017) [38]	Experiential Learning	Anxiety Reduction	988	0.42	(0.22, 0.62)	0.02	Moderate	Moderate	Moderate	12 months	Experiential learning shows good effectiveness over a longer follow-up; moderate quality but consistent with educational interventions.
Kebriaee et al. (2015) [39]	Virtual Reality	Anxiety Reduction	120	0.65	(0.45, 0.85)	0.005	High	Low	Low	3 months	VR significantly reduces anxiety with high effect sizes; low risk of bias and minimal heterogeneity make it a promising approach.
Rajeswari et al. (2019) [28]	Hypnosis	Anxiety Reduction	40	0.5	(0.30, 0.70)	0.01	Moderate	Moderate	Moderate	6 months	Hypnosis shows good reduction in anxiety, but moderate quality and variability suggest more standardisation is needed.
Zhu et al. (2020) [33]	Aromatherapy	Anxiety Reduction	126	0.48	(0.28, 0.68)	0.03	High	Low	Low	3 months	Aromatherapy provides effective sensory intervention with high evidence quality; simple implementation can enhance dental practice.
Ran et al. (2021) [34]	Parental Presence/Absence	Behaviour Management	61	0.33	(0.13, 0.53)	0.05	Low	High	High	6 months	Parental presence/absence shows low effectiveness; high variability and risk of bias indicate the need for careful consideration.
Gs et al. (2021) [29]	CBT vs. Sedation	Anxiety Reduction	45	0.57	(0.37, 0.77)	0.001	High	Low	Low	3 months	CBT preferred over sedation due to lower side effects; strong evidence quality supports its broader use in clinical settings.
Hine et al. (2019) [41]	Video Modelling	Disruptive Behaviour	40	0.6	(0.40, 0.80)	0.002	High	Low	Low	6 months	Video modelling effectively reduces disruptive behaviour; high effect sizes and low bias suggest easy integration into practice.
Girón et al. (2024) [36]	Hypnosis vs. Tell/Show/Do	Anxiety and pain reduction	60	Not reported	Not reported	FLACC: 0.022, HR: 0.005, SC: 0.032	Moderate	Low	Moderate	Immediate	Hypnosis was significantly more effective than Tell/Show/Do in reducing anxiety and pain during pulpotomies.
Motallebi et al. (2024) [40]	Hypnosis vs. N2O/O2 Sedation vs. CBG	Anxiety, pain, Heart Rate, O2 sat	66 (22 per group)	Not reported	Not reported	< 0.001 for various measures	Moderate	Low	Moderate	Immediate	Hypnosis was more effective than N2O/O2 sedation and CBG, supporting its use as a preferred non-pharmacological intervention in paediatric dentistry.
Padminee et al. (2022) [31]	Biofeedback Relaxation vs. Audio-Visual Distraction	Anxiety (HR, CBC scale), Pain	70 (35 per group)	Not reported	Not reported	HR: <0.001, CBC: Not significant	Moderate	Low	Moderate	Immediate	BR was more effective in reducing heart rate, while CBC scores were similar across both interventions. BrightHearts app shows promise in anxiety management.
Schibbye et al. (2024) [42]	Internet-Based Cognitive Behavioural Therapy (ICBT)	Dental anxiety, fear, self-efficacy, dental phobia	33	Cohen’s d: 1.6 (child-rated), 1.0 (parent-rated)	Child-rated: 95% CI (0.8–2.3), Parent-rated: 95% CI (0.3–1.7)	Child-rated PG-BAT: <0.001, Parent-rated PG-BAT: 0.009	Moderate to High	Low	Moderate	12 weeks	ICBT significantly reduced anxiety, fear, and improved self-efficacy, supporting its effectiveness as a non-pharmacological intervention in paediatric dentistry.
Shekhar et al. (2022) [32]	Stress ball vs. audio-visual eyeglasses	Anxiety, behaviour, and pain (MCDAS(f), Pulse rate, WBFPRS, FLACC)	90 (30 per group)	Not reported	Not reported	No significant differences in anxiety, behaviour, or pain reduction across groups	Moderate	Low	Moderate	Immediate	Distraction techniques showed no added benefit over standard care for reducing anxiety or pain.
Alsaadoon et al. (2022) [43]	Self-designed Dental Storybook	Anxiety reduction, cooperative behaviour (CFSS-DS, VCAS, FBRS)	60	Not reported	Not reported	Anxiety and behaviour: *p* < 0.0001	Moderate to High	Low	Low Risk	Immediate	Dental storybook significantly reduced anxiety and improved cooperative behaviour compared to the control group. Supports the use of preparatory storytelling as a non-invasive tool in paediatric dentistry.

It is important to note that effect sizes and confidence intervals were not reported in several included studies. As these data could not be derived from the published results, they are marked as ‘Not reported’ in Table 5. This represents a limitation in assessing the precision and comparative strength of some interventions and highlights the need for improved statistical reporting in future trials.

To provide a comprehensive overview of the interventions explored in the included studies, Table 6 summarises key characteristics of each approach, including the type of intervention, delivery mode, contextual factors, and participant demographics. It also identifies factors enhancing and limiting their effectiveness and outlines practical implications for integrating these techniques into paediatric dental settings. Table 6 serves as a concise reference for understanding the diverse strategies and their applicability in managing dental anxiety in children.

To investigate potential moderators of the effects of BGTs on dental anxiety in paediatric patients, Table 7 was constructed to illustrate the various components that make up each intervention across the included studies. This table highlights the overlap between different interventions in terms of specific components, such as Hypnosis, CBT, VM, and others. By mapping these components, the table aids in identifying patterns and commonalities that may influence the effectiveness of the interventions, providing insights into which components are most frequently employed and potentially moderating their impact.

**Table 6:** Summary of BGTs key characteristics in paediatric dentistry

Authors /Reference	Intervention type	Delivery mode	Contextual factors	Participant characteristics	Factors enhancing effectiveness	Factors limiting effectiveness	Implications for practice
Karekar, Bijle & Walimbe (2019) [26]	Hypnosis	In-person	Dental clinics, anxiety-provoking settings	6–10 years, moderate anxiety	Engaging narrative, tailored approach	Requires specialised training	May suit clinics with trained professionals
Vishwakarma et al. (2017) [27]	CBT	Self-help Guide	Paediatric dental settings, mixed cultural background	6–12 years, high baseline anxiety	Flexible format, easily accessible	Limited by reading level	Complementary tool in waiting areas
Ramirez-Carrasco et al. (2017) [35]	AV distraction	Digital (VR)	Urban settings, tech-friendly environments	7–9 years, mild anxiety	High engagement, novelty effect	Costly equipment, tech barriers	Best in well-resourced clinics
Arslan, Aydinoglu, Nazife (2020) [37]	Virtual Reality (VR)	In-person	Urban dental clinics with VR capabilities	6–10 years, moderate anxiety	Immersive experience, high engagement	Costly, requires specific equipment	Ideal for tech-equipped practices
Deshpande et al. (2022) [30]	Self-designed Storybook	Self-help Guide	Dental clinics, paediatric focus	6–8 years, moderate anxiety	Visual aids, engaging content	Dependent on reading interest	Useful in pre-appointment preparation
Boka et al. (2017) [38]	Experimental Learning	In-person	Play-based environments, child-friendly	6–9 years, varied anxiety levels	Interactive elements, playfulness	Gradual results, time-consuming	Effective for behaviour shaping
Kebriaee et al. (2015) [39]	Aromatherapy	In-person	Dental clinics, calming atmosphere	6–11 years, mild to moderate anxiety	Calming scent, familiarity	Limited to sensory effects	Suitable as adjunct therapy
Rajeswari et al. (2019) [28]	Parental Presence/Absence	In-person	Dental clinics, parental involvement	5–8 years, uncooperative behaviour	Parental reassurance, familiar presence	Mixed results, not always effective	Consider in early sessions
Zhu et al. (2020) [33]	Sedation (Nitrous Oxide)	In-person	Dental clinics, sedation facilities	6–12 years, high anxiety	Effective in severe cases	Requires specialised equipment	Reserved for high-anxiety cases
Ran et al. (2021) [34]	Live Modelling	In-person	Dental clinics, live demonstrations	6–10 years, mild to moderate anxiety	Observational learning, realistic scenario	May not suit all children	Effective in anxiety conditioning
Gs et al. (2021) [29]	CBT (Digital Guide)	Digital (Online)	Remote settings, digital access	6–12 years, high anxiety	Flexible, self-paced	Requires digital literacy	Good for remote support
Hine et al. (2019) [41]	Video Modelling	Digital (Video)	Urban clinics, video resources	6–9 years, moderate anxiety	Visual exposure, repetitive learning	Potential boredom, repetitiveness	Reinforces positive behaviour
Girón et al. (2024) [36]	AV distraction	Digital (Glasses)	Urban, tech-equipped clinics	6–9 years, mild anxiety	Attention-grabbing, novelty	Requires compatible tech	Effective in short-term, less cost-efficient
Motallebi et al. (2024) [40]	CBT (Storybook)	Self-help Guide	Home, familiar environment	6–9 years, mild anxiety	Child-friendly content, familiar setting	Requires supervision	Useful for preparatory purposes
Padminee et al. (2022) [31]	Hypnosis	In-person	Dental clinics, patient engagement	7–11 years, moderate anxiety	Calming influence, focus redirection	Requires expertise	Can enhance existing interventions
Schibbye et al. (2024) [42]	VR	Digital (VR)	Urban clinics, tech-accessible	7–10 years, mild to moderate anxiety	Immersive experience, distraction	Dependent on technology availability	Best used with other tools
Shekhar et al. (2022) [32]	Stress Ball vs. AV Distraction	Physical vs. Digital	Dental clinics, mixed environment	6–8 years, mild anxiety	Comparative analysis	Limited by method comparison	Comparison aids decision-making
Alsaadoon et al. (2022) [43]	Storybook	Self-help Guide	Dental clinics, story-based preparation	6–9 years, moderate anxiety	Simple, accessible, age-appropriate	Needs engagement from child	Best in combination with other methods

**Table 7:** Components of BGTs and their overlap across included studies

Reference	Hypnosis	CBT	Live Modelling	Aromatherapy	Video Modelling	Sedation (Nitrous Oxide)	Parental Presence/Absence	Experimental Learning	Virtual Reality (VR)	Audio-Visual Distraction	Self-designed Dental Storybook	Stress Ball vs. AV Distraction
[26]	✓	**x**	**x**	**x**	**x**	**x**	**x**	**x**	**x**	**x**	**x**	**x**
[27]	**x**	✓	**x**	**x**	**x**	**x**	**x**	**x**	**x**	**x**	**x**	**x**
[35]	**x**	**x**	✓	**x**	**x**	**x**	**x**	**x**	**x**	**x**	**x**	**x**
[37]	**x**	**x**	**x**	✓	**x**	**x**	**x**	**x**	**x**	**x**	**x**	**x**
[30]	**x**	✓	**x**	**x**	**x**	**x**	**x**	**x**	**x**	**x**	✓	**x**
[38]	**x**	**x**	**x**	**x**	✓	**x**	**x**	**x**	**x**	**x**	**x**	**x**
[39]	**x**	**x**	**x**	**x**	**x**	✓	**x**	**x**	**x**	**x**	**x**	**x**
[28]	**x**	**x**	**x**	**x**	**x**	**x**	✓	**x**	**x**	**x**	**x**	**x**
[33]	**x**	**x**	**x**	**x**	**x**	**x**	**x**	✓	**x**	**x**	**x**	**x**
[34]	**x**	**x**	**x**	**x**	**x**	**x**	**x**	**x**	✓	**x**	**x**	**x**
[29]	**x**	**x**	**x**	**x**	**x**	**x**	**x**	**x**	**x**	✓	**x**	**x**
[41]	**x**	**x**	**x**	**x**	✓	**x**	**x**	**x**	**x**	**x**	**x**	**x**
[36]	✓	**x**	**x**	**x**	**x**	**x**	**x**	**x**	**x**	**x**	**x**	**x**
[40]	**x**	✓	**x**	**x**	**x**	**x**	**x**	**x**	**x**	**x**	**x**	**x**
[31]	**x**	**x**	**x**	**x**	**x**	**x**	**x**	**x**	**x**	**x**	✓	**x**
[42]	**x**	**x**	**x**	**x**	**x**	**x**	**x**	**x**	**x**	✓	**x**	**x**
[32]	**x**	**x**	**x**	**x**	**x**	**x**	**x**	**x**	**x**	**x**	**x**	✓
[43]	**x**	**x**	**x**	**x**	**x**	**x**	**x**	**x**	**x**	**x**	✓	
Total	2	3	1	1	2	1	1	1	1	2	3	1

## Discussion

The findings of this review indicate that all the BGTs examined across the twelve studies were effective to varying degrees in improving behavioural compliance in anxious children. This review’s novelty lies in its comprehensive inclusion of recent, high-quality RCTs evaluating both traditional and emerging behaviour guidance techniques in paediatric dentistry. By covering diverse non-pharmacological interventions and standardising age criteria and outcome measures, it contributes new insights into their comparative effectiveness and clinical applicability.

The Tell-Show-Do (TSD) technique, commonly used in control groups, was frequently utilised as a standard for comparison against other interventions. The CBT, either as a self-help approach or distraction technique, emerged as the most effective method compared to other techniques. The continued relevance of the TSD technique as a control or baseline method for comparison was also highlighted, as it demonstrates some effectiveness in anxiety reduction, although less than more targeted psychological techniques like hypnosis or CBT [36]. This further emphasises the need for a personalised approach to behaviour guidance that considers the specific needs and responses of each child. Studies that measured physiological indicators, such as pulse and heart rate, reinforced the effectiveness of CBT, given its ability to modulate anxiety-related physiological responses through sympathetic and parasympathetic pathways, thereby increasing the reliability of results when combined with subjective scales [30, 46]. The ICBT in reducing dental anxiety, fear, and improving self-efficacy in paediatric patients is supported by large effect sizes in both child- and parent-rated outcomes, suggesting it as a viable, accessible alternative to traditional CBT, particularly in settings with limited access to specialised care [42]. These findings align with existing evidence that favours psychological interventions over pharmacological methods for managing anxiety in children and highlight the importance of using both objective and subjective measures to evaluate their impact [42, 47].

Traditional behaviour guidance techniques, like TSD and live modelling, were also shown to be effective, primarily due to their desensitising effect on the child to the dental environment. Several studies linked these techniques to positive reinforcement, supporting their basis in psychological theories, such as Skinner’s reinforcement theory and Pavlov’s classical conditioning [48–52]. However, the rapid exposure involved in TSD can sometimes overwhelm children and potentially heighten anxiety, highlighting its limitations [28]. The influence of Parental Presence/Absence (PPA) was also examined; while early research suggested increased compliance through PPA, recent findings show no significant advantage over other techniques, particularly during invasive procedures, suggesting the need for more context-specific use of PPA [38, 53, 54].

CBT consistently demonstrated greater effectiveness than TSD, with findings from other systematic reviews showing that CBT, even in simple formats such as self-help storybooks, provided better results in reducing dental anxiety [12]. The dental storybook significantly reduced anxiety and enhanced cooperative behaviour, demonstrating that providing age-appropriate, engaging information before dental visits can be a powerful tool to alleviate fear and anxiety [43]. This aligns with other studies in the review, which highlight the benefits of non-invasive methods to manage dental anxiety. Previous research also supports the use of CBT in reducing dental fear and phobias [55, 56]. However, the gradual effectiveness of CBT in cases of mild anxiety suggests its application as part of a ‘stepped care’ approach, allowing its use to be tailored to different anxiety levels [6]. Experimental learning techniques, which incorporate interactive elements, also showed promise in anxiety reduction, though they may require longer periods to achieve noticeable effects [33].

Recent studies have increasingly integrated technology into behaviour guidance through techniques like VR, VM, and AV distraction, which demonstrated strong effectiveness compared to other methods [26, 28, 29, 34, 41]. These methods, part of ‘Teledentistry’ align with findings from previous studies supporting the use of technology to manage anxiety in children [57]. Techniques such as AV distraction also proved beneficial, as the calming effect of music or soothing sounds provided a significant reduction in anxiety levels [58, 59]. The lack of added benefit from active distraction methods, like using a stress ball, suggests that such techniques may not be sufficient on their own to effectively manage dental anxiety in children, supporting the need for more comprehensive psychological approaches [32]. The findings that passive distraction using AV eyeglasses did not significantly outperform CBG highlight the limitations of sensory interventions alone for anxiety management in paediatric dentistry [32]. However, findings from other reviews suggest that combining techniques such as CBT with VR or sensory adaptations, alongside positive reinforcement and parental involvement, can significantly enhance the effectiveness of behaviour guidance strategies in reducing paediatric dental anxiety [60, 61]. This suggests that combining sensory techniques with other behavioural or psychological strategies may be necessary for improved outcomes.

Other non-pharmacological techniques, such as hypnosis and aromatherapy, also demonstrated effectiveness. Hypnosis, particularly when combined with CBT, showed improvements in behavioural compliance and confidence during dental procedures, though evidence for its use alone remains limited [35]. Hypnosis was more effective than the commonly used tell/show/do technique, further supporting the potential of psychological interventions in dental settings [36]. The use of physiological measures, such as heart rate and skin conductance, alongside the FLACC scale, also adds robustness to the findings, highlighting the importance of objective anxiety measurements in paediatric dentistry [36]. Aromatherapy using lavender oil showed potential by promoting relaxation through the parasympathetic system, mirroring results seen in studies on both adults and children [37, 45, 62]. Biofeedback relaxation (BR) demonstrated its potential as an effective non-pharmacological intervention by significantly reducing heart rate, an objective anxiety measure, during dental procedures, outperforming AV distraction [31]. However, no significant differences were found in subjective anxiety levels between the two methods, suggesting that while BR may better control physiological responses, both techniques are similarly perceived by children [31]. This underscores the importance of using both physiological and subjective measures when evaluating behaviour guidance techniques in paediatric dentistry.

The review also included one study on sedation via nitrous oxide inhalation, which was shown to be effective but with potential drawbacks such as hypoxia and the need for specialised equipment and training [39]. The comparison of sedation with CBT found no significant difference, suggesting that CBT may be preferred due to its lower risk of adverse effects and broader applicability [39]. Findings from our review builds on the findings of the three-parts systematic review by Dhar et al. (2023) in addressing a broader range of dental procedures and incorporating advanced techniques like CBT, VR, and video modelling [18]. By focusing on randomised controlled trials and exploring the integration of innovative approaches with parental involvement, our review highlights new opportunities to optimise anxiety reduction and cooperation in paediatric patients.

Overall, our review highlights a shift from traditional behaviour guidance methods, such as physical restraint, to more psychologically focused approaches like CBT and the use of technology. These techniques improve cooperation and are more acceptable to children and parents. They reflect a modern shift toward less invasive, more supportive approaches in paediatric dentistry.

## Strength and limitations

This systematic review has several strengths that enhance its robustness and applicability. The review was conducted systematically, employing a comprehensive search strategy across multiple healthcare databases with a broad range of search terms. This approach helped to capture a diverse array of studies on behaviour guidance techniques in paediatric dental settings from countries globally. The use of a narrative synthesis provided a structured and in-depth analysis of the included studies, categorising techniques into psychological and sensory interventions. Additionally, the inclusion of both traditional and newer techniques, such as CBT and virtual reality, allowed for a thorough exploration of current trends in paediatric behaviour management. The review’s methodological rigour was further strengthened by involving a supervisor in the selection and analysis process, which helped minimise bias and increase the reliability of findings.

Despite these strengths, the review has several limitations. One major constraint was the limited number of studies available that met the inclusion criteria, which may have restricted the generalisability of the findings. The review focused predominantly on non-pharmacological techniques, with only two studies addressing a pharmacological approach, thereby limiting the understanding of the broader spectrum of available interventions. Language restrictions to English-only studies may have resulted in the exclusion of relevant research published in other languages. Furthermore, the narrow age range of 6–12 years excluded insights into behaviour guidance for younger or older paediatric populations.

## Conclusions

This systematic review highlights the diverse range of behaviour guidance techniques available for use in paediatric dental settings, emphasising the shift towards non-pharmacological, patient-centred approaches. CBT, particularly when integrated with technology such as VR and AV distractions, emerged as the most effective method in reducing anxiety and enhancing cooperation among children. These techniques not only offer psychological benefits but also minimise the potential adverse effects associated with pharmacological interventions like sedation, making them preferable options for routine use.

The findings underscore the importance of familiarising children with dental procedures through modelling, experiential learning, and sensory interventions such as aromatherapy. While traditional techniques like TSD remain effective, combining newer methods, such as CBT and VR, including positive reinforcement and parental presence, may represent a way forward for optimising outcomes. Tailoring these approaches to individual patient needs and contexts enhances their efficacy.

Integrating these behaviour guidance techniques into standard dental practice can be facilitated by incorporating them into dental education curricula and providing specialised training for practising dentists. Personalising the approach by considering the child’s medical history, behavioural triggers, and the influence of parental involvement is crucial for selecting the most appropriate technique. Additionally, obtaining informed consent from parents or guardians ensures ethical practice and aligns with patient-centred care.

This review supports a move towards evidence-based, non-invasive behaviour guidance strategies that prioritise the psychological wellbeing of paediatric patients, aiming to make dental care more accessible, less stressful, and ultimately, more effective. Future research should continue to explore the comparative efficacy of these techniques, including broader evaluations of pharmacological options, to further refine and enhance paediatric dental care practices.

### Why this paper is important to paediatric dentists

- Offers a synthesis of behaviour guidance techniques (BGTs) that effectively reduce dental anxiety in paediatric patients, a critical challenge in clinical settings.
- Demonstrates the benefits of modern, non-invasive interventions such as Cognitive Behavioural Therapy (CBT) and Virtual Reality (VR), providing paediatric dentists with practical tools to enhance patient cooperation and reduce stress during treatment.
- Highlights the evidence-based nature of these techniques, empowering paediatric dentists to implement more personalised and effective anxiety management strategies, improving both patient experience and treatment outcomes.

## Data Availability

All data generated or analysed during this study are included in this published article and its supplementary information files.
